# The Link between Gaucher Disease and Parkinson’s Disease Sheds Light on Old and Novel Disorders of Sphingolipid Metabolism

**DOI:** 10.3390/ijms20133304

**Published:** 2019-07-05

**Authors:** Rossella Indellicato, Marco Trinchera

**Affiliations:** 1 Department of Health Science, University of Milan, 20142 Milano, Italy; 2 Department of Medicine and Surgery, University of Insubria, 21100 Varese, Italy

**Keywords:** autophagy, ganglioside, lysosome, rare disease

## Abstract

Sphingolipid metabolism starts with the biosynthesis of ceramide, a bioactive lipid and the backbone for the biosynthesis of complex sphingolipids such as sphingomyelin and glycosphingolipids. These are degraded back to ceramide and then to sphingosine, which enters the ceramide–sphingosine-1-phosphate signaling pathway or is further degraded. Several enzymes with multiple catalytic properties and subcellular localizations are thus involved in such metabolism. Hereditary defects of lysosomal hydrolases have been known for several years to be the cause of lysosomal storage diseases such as gangliosidoses, Gaucher disease, Niemann–Pick disease, Krabbe disease, Fabry disease, and Farber disease. More recently, many other inborn errors of sphingolipid metabolism have been recognized, involving enzymes responsible for the biosynthesis of ceramide, sphingomyelin, and glycosphingolipids. Concurrently, epidemiologic and biochemical evidence has established a link between Gaucher disease and Parkinson’s disease, showing that glucocerebrosidase variants predispose individuals to α-synuclein accumulation and neurodegeneration even in the heterozygous status. This appears to be due not only to lysosomal overload of non-degraded glucosylceramide, but to the derangement of vesicle traffic and autophagy, including mitochondrial autophagy, triggered by both sphingolipid intermediates and misfolded proteins. In this review, old and novel disorders of sphingolipid metabolism, in particular those of ganglioside biosynthesis, are evaluated in light of recent investigations of the link between Gaucher disease and Parkinson’s disease, with the aim of better understanding their pathogenic mechanisms and addressing new potential therapeutic strategies.

## 1. Introduction

**Figure 1:**
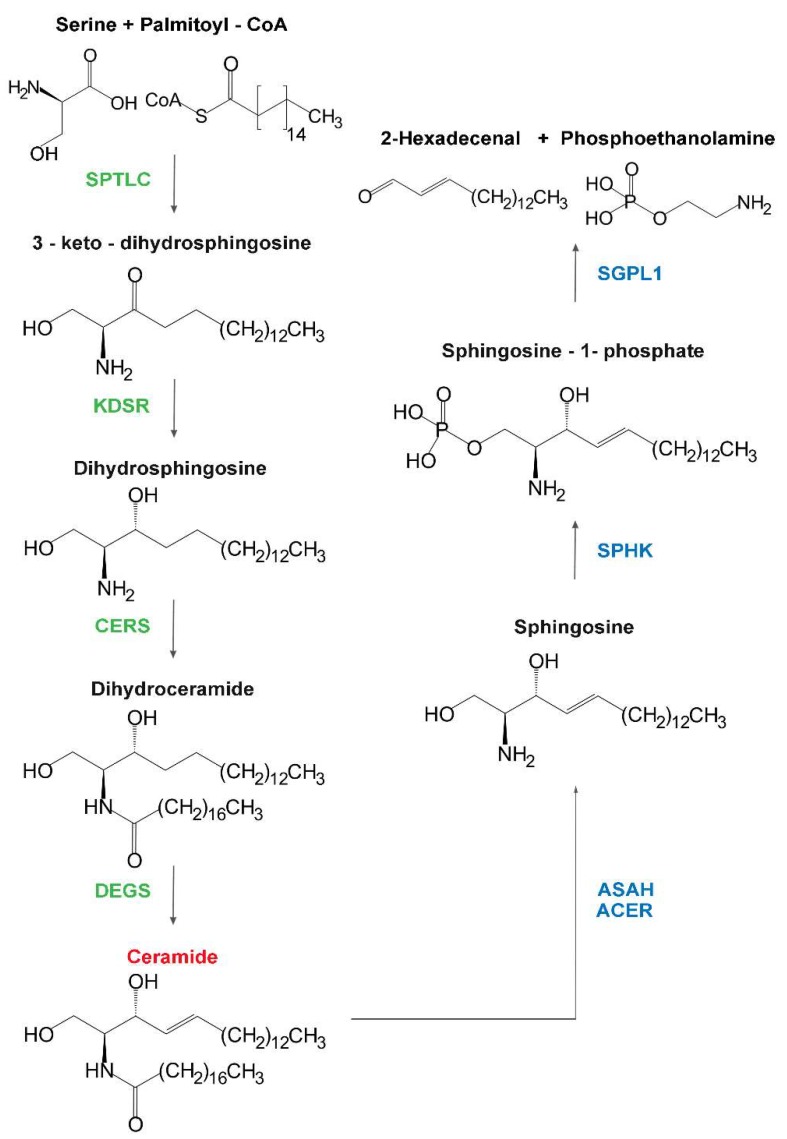
Biosynthesis and degradation of ceramide. SPTLC, serine palmitoyl transferase; KDSR, 3-keto-dihydro-sphingosine reductase; CERS, dihydroceramide synthase; DEGS, dihydroceramide desaturase; ASAH, acid ceramidase; ACER, alkaline ceramidase; SPHK, sphingosine kinase; SGPL1, sphingosine lyase. For simplicity, only stearic acid is depicted as the acyl chain of ceramides, but longer fatty acids can be added by different CERSs.

**Figure 2:**
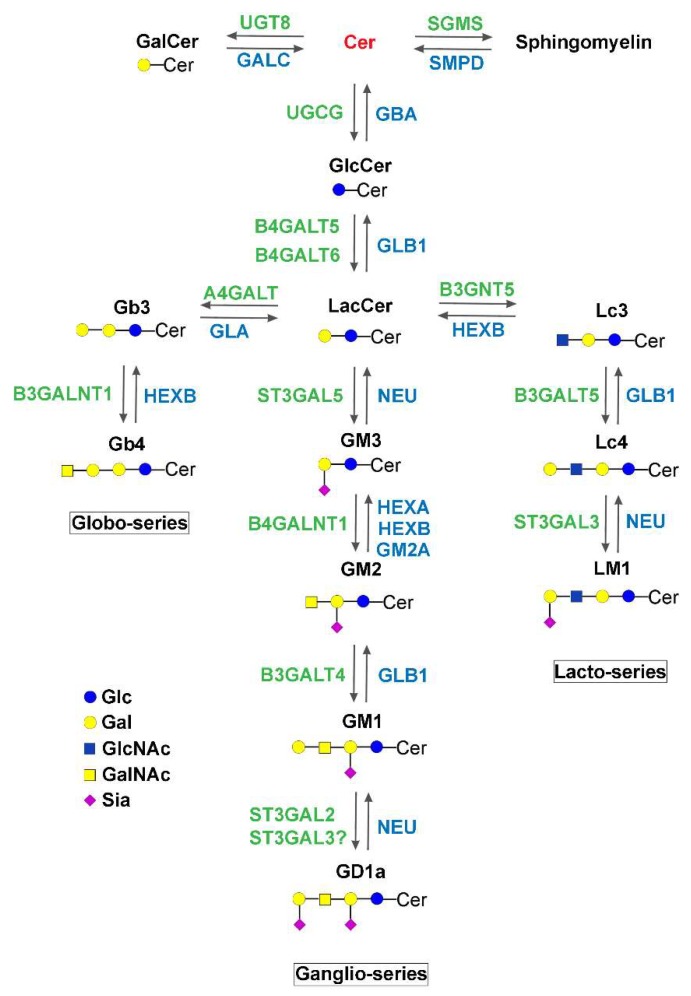
Biosynthesis and degradation of complex glycosphingolipids. Monosaccharides are depicted according to the current representation: Glc, glucose; Gal, galactose; GlcNAc, N-acetylglucosamine; GalNAc, N-acetylgalactosamine; Sia, sialic acid. Enzyme symbols are according to the HUGO nomenclature (see Table 2; Table 3 for enzyme details). UGT8, GalCer synthase; GALC, galactocerebrosidase; SGMS, sphingomyelin synthase; SMPD, sphingomyelinase; UGCG, GlcCer synthase; GBA, glucocerebrosidase; B4GALT, β1,4-galactosyltransferase; GLB1, β-galactosidase; A4GALT, α1,4-galactosyltransferase; GLA, α-galactosidase; B3GALT, β1,3-GalNActransferase; Hex, hexosaminidase; B3GNT, β1,3-GlcNAc transferase; B3GALT, β1,3-galactosyltransferase; ST3GAL, galactoside-α2,3-sialyltransferase; NEU, neuraminidase (sialidase). Lc3, lactotriaosylceramide GlcNAcβ1,3Galβ1,4GlcCer; Lc4, lactotetraosylceramide; LM1, sialyllactotetraosylceramide, Siaα2,3Galβ1,3GlcNAcβ1,3Galβ1,4GlcCer; Gb3, globotriaosylceramide Galα1,3Galβ1,4GlcCer; Gb4, globotetraosylceramide GalNAcβ1,3Galα1,3Galβ1,4GlcCer.

Sphingolipid metabolism starts in the endoplasmic reticulum (ER) with the condensation of serine and palmitic acid, producing 3-keto-dihydrosphingosine (also named 3-keto-sphinganine), and it continues through successive enzymatic reactions leading to the synthesis of ceramide [1] (Figure 1). Ceramide plays several roles in cell homeostasis, itself acting as a regulator or through the generation of other bioactive lipids such as ceramide-1-phosphate and sphingosine-1-phosphate (S1P) [2]. Nevertheless, a large amount is used as the backbone for the biosynthesis of sphingomyelin (SM) and glycosphingolipids (GSLs) (Figure 2). In particular, ceramide is acted upon by UGT8, galactosylceramide (GalCer) synthase (see Table 1, Table 2 and Table 3 for enzyme symbols) in the ER [1], and SGMS, SM synthase [3] or UGCG, glucosylceramide (GlcCer) synthase [4] in the Golgi apparatus. GlcCer may undergo many different further glycosylations, giving rise to a plethora of GSLs, depending on the specific glycosyltransferase machinery present in the various cell types. These include gangliosides, globosides, and other neutral compounds (Figure 2). Ceramide, SM, and GSLs flow through the Golgi to the plasma membrane or other organelles (such as mitochondria in the case of ceramide) via vesicular-mediated [5] or protein-mediated traffic [6]. The plasma membrane is considered the main destination of such compounds, which may also be delivered outside the cell in the form of microvesicles [7] or channeled along the endocytic pathways toward phagosomes and lysosomes. In the lysosomes, complex sphingolipids are stepwise degraded to fatty acids and sphingosine (Figure 1 and Figure 2). Sphingosine reaches the ER, where it enters the Cer–S1P signaling pathway. S1P can be also degraded to phosphoethanolamine and 2-hexadecenal [1] (Figure 1). Defects of specific lysosomal hydrolases (Table 1) involved in this process have been known for several years and constitute the main group of lysosomal storage disorders due to inborn errors of metabolism [8]. These include the gangliosidoses, Niemann–Pick disease, Fabry disease, Krabbe disease, Gaucher disease, and Farber disease. More recently, defects of several enzymes responsible for the biosynthetic steps (Table 2 and Table 3) were found to be associated with human diseases: these include variants of serine palmitoyl transferases (SPTLC) [9,10], 3-keto-dihydro-sphingosine reductase (KDSR) [11,12,13], dihydro-ceramide synthases (CERS) [14,15,16,17,18], dihydroceramide desaturase (DEGS) [19,20,21], sphingomyelin synthases (SGMS) [22,23], GlcCer synthase (UGCG) [24], GM3 synthase (ST3GAL5), GM2/GD2/GA2 synthase (B4GALNT1), and CMP-Sial: GlcNAcβ1,3(4) sialyltransferase (ST3GAL3) [25]. The latter four enzymes are glycosyltransferases and, thus, the related diseases belong to the wide family of the congenital disorders of glycosylation (CDG) [26]. It is worth noting that some enzymes of sphingolipid metabolism exist in different isoforms representing different gene products, sometimes with different subcellular localization, as in the case of ceramidases, ceramide synthases, sphingomyelinases, and SM synthases (Table 1, Table 2 and Table 3). It is not surprising that disorders involving different isoforms may produce very different clinical phenotypes. Variants of CERS1 and -2 result in a myoclonus epilepsy [14,15,17], while CERS3 variants determine a form of ichthyosis [16]. Glucocerebrosidase (GBA) variants are responsible for Gaucher disease (GD) [27], while GBA2 variants result in hereditary spastic paraplegia 46 (HSP46) [28]. Acid ceramidase ASAH1 deficiency is the cause of Farber disease [29], while alkaline ceramidase (ACER3) variants are responsible for progressive leucodystrophy [30]. Many of such disorders result in extremely heterogeneous clinical syndromes, in terms of both symptoms and severity, and some give rise to clinical features overlapping those of disorders involving totally unrelated genes [31]. GD is the most relevant example of the former. The clinical presentation ranges from a mild visceral impairment detectable in adulthood to a severe neurologic impairment that is lethal by early childhood, without a clear relationship with the underlying mutation or the amounts of residual enzyme activity [27,32,33]. On the other hand, B4GALNT1-CDG is one of about 70 known genetic defects determining an inherited spastic paraplegia [34], and ST3GAL3-CDG is reported as one of the many conditions causing West syndrome, potentially evolving to Lennox–Gestaut syndrome [35]. Many such disorders are typically transmitted in an autosomal recessive manner (see Table 1, Table 2 and Table 3). However, emerging data show that heterozygous carriers of glucocerebrosidase GBA [33], sphingomyelinase SMPD1 [36], galactocerebrosidase (GALC) [37], and α-galactosidase (GLA) [38] variants are as much at risk for neurodegenerative diseases such as synucleopathies and multiple sclerosis as those with recessive homozygotes.

The purpose of this review is to evaluate recent data concerning disorders of sphingolipid metabolism where the pathogenic role played by the loss of function of enzymes appears unable to explain the whole picture, while other mechanisms seem to cooperate with the clinical phenotype. Among them, the emerging candidates include the impairment of vesicle traffic in the context of autophagy/lysosome function and altered trafficking and metabolism of the variant proteins. In this regard, the data on GD as the main risk factor for Parkinson’s disease (PD) suggest that the GD/PD connection may represent a pathogenic paradigm able to shed light on other disorders of sphingolipid metabolism. In particular, we focus on those affecting ganglioside biosynthesis as the disorders where such aspects could be relevant. This is interesting not only to better understand sphingolipid biology and the disease pathogenesis, but to address novel potential therapeutic strategies.

## 2. GBA Variants, GD, PD, and How They Interplay

**Figure 3:**
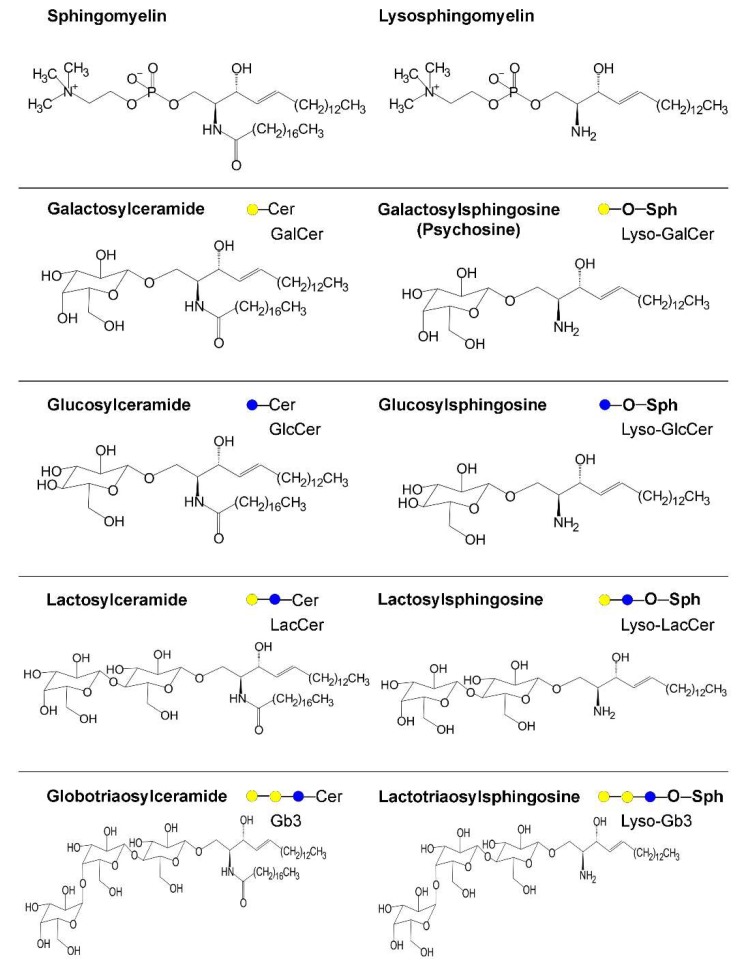
Structure of lysosphingolipids and related compounds. Lysosphingolipds are complex sphingolipids deacylated by the action of acid ceramidase on the corresponding compounds accumulated because of an inborn error of metabolism. Sph, sphingosine. For simplicity, only stearic acid is depicted as the acyl chain, but longer fatty acids are also frequently present.

GD is a lysosomal storage disorder caused by recessive mutations in the *GBA* gene, coding the β-galactosidase responsible for the degradation of GlcCer to glucose and ceramide [39]. Reduced GBA activity leads to the accumulation of GlcCer and/or its immediate by-product glucosylsphingosine (GlcSph), formed by the action of acid ceramidase on GlcCer (Figure 3). GBA is synthesized in the ER as a 536- or 516-amino-acid protein, containing a short signal peptide of 19 or 39 amino acids at the N-terminus that is trimmed soon after translation [40]. The residual 497 amino acid peptide is then glycosylated [41] and recognized by the SCARB2 (scavenger receptor class B member 2) system [42]. The GBA/SCARB2 complex [43] experiences traffic through the Golgi apparatus and endocytic vesicles and then reaches the lysosomes, which are the sites of action. In the lysosomes, GBA activity is also controlled by the activator protein saponisin C. Another glucocerebrosidase is coded by the human genome, GBA2, which has a microsomal localization and is not involved in GD but instead is responsible for a form of HSP, reported as HSP46 [28,44] (Table 1). From a clinical point of view, patients suffering GD present two main distinct syndromes [8,27]. Type 1 GD is characterized by the involvement of several visceral organs without that of the central nervous system, while types 2 and 3 present serious neurologic symptoms and are defined as neuronopathic GD. Symptoms of type 1 disease appear in adulthood and classically include anemia, hepatosplenomegaly, bone pain, and other inflammatory signs. The inflammatory effect of GlcCer accumulation was recently proposed to be the consequence of complement C5a activation [45]. However, more recent reports identified patients with type 1 GD developing peripheral polyneuropathy at an older age [33]. Moreover, a subset of patients was recently reported to present mild but distinctive intellectual impairment, suggesting an involvement of the central nervous system [46,47]. Conversely, neuronopathic disease is much more severe: early onset of neurologic symptoms includes supranuclear gaze palsy, cognitive impairment (frequently, features of oppositional defiant disorder), seizures, various muscular problems, and even sudden death. Rapid progression of the disease with death in early childhood is characteristic of type 2 disease, while in type 3 the progression is slower. Type 2 and 3 diseases are also suggested to be the more evident forms of a spectrum disease [33]. About 300 different mutations have been reported to affect the *GBA* gene. Some of them, such as L444P and N370S, are more frequent and account for several cases. There is a wide variety of mutation frequency between ethnicities. For instance, up to 1 in every 15 persons is considered a carrier of a *GBA* mutation in the Ashkenazi Jewish population, versus 1 in every 100 for others [27,48]. Despite several attempts to find a correlation between individual mutations and the clinical syndrome, none have been firmly established so far, and patients affected by the same mutation present a large variability of symptoms [27,32]. Data of the residual enzyme activity and its potential role as a predictor of disease severity are also contradictory [33,49]. Both the common L444P and N370S variants are associated with a similar strong reduction in enzyme activity, about 80–90% compared with the wild type enzyme [50]. Conversely, the N370S variant is more frequently associated with the type 1 disease and a mild or very mild phenotype, while the L444P variant is associated with type 3 disease and a severe neuronopathic phenotype in general. Some reports indicate that GBA variants are instable or misfolded proteins, which do not undergo normal intracellular traffic, irrespective of the amount of catalytic activity maintained [39,50], suggesting a role for proteostasis in GD. A very intriguing issue in this field is the epidemiological evidence, established about 10 years ago, that *GBA* mutations are the major genetic risk factors for the development of PD in elderly people [51]. Such a risk is associated not only with GD but with the carrier status of heterozygous *GBA* variants, as proven in the family members of GD patients. The molecular mechanism linking GBA mutations to PD appears complex. Experimental evidence indicates that reduced GBA activity and concurrent accumulation of GlcCer takes places in normal aging of the mouse brain [52]. In humans, reduced expression and activity of GBA was found in the substantia nigra of the post-mortem brains of patients suffering from PD, both sporadic and associated with GBA mutations [53]. Pathologically, the hallmark of PD is the presence of Lewy bodies in affected cells, consisting of the accumulation of α-synuclein aggregates in the cells. The derangement of α-synuclein metabolism in PD is suspected to be the consequence of impaired autophagy and lysosome clearance [50,54]. The interaction between GBA variants, with or without loss of GBA activity, and α-synuclein accumulation has been intensively studied in neuronal cultures and in animal models. Deposition of α-synuclein aggregates impairs vesicle trafficking [55], which disrupts traffic of GBA and other hydrolases to the lysosomes, favoring further α-synuclein accumulation and self-maintenance of the pathogenic process [54]. Accordingly, double transgenic mice heterozygous for a null GBA allele and expressing human α-synuclein showed a relevant disruption of dopaminergic neurons associated with the accumulation of GlcSph but not GlcCer [56]. Comparable results were found by analyzing post-mortem brains of patients suffering sporadic PD or dementia with Lewy bodies, where the levels of α-synuclein were inversely correlated with those of the GBA protein and directly with those of GlcSph [57]. On this basis, it was hypothesized that parallel reduction of GlcCer synthase activity, with concurrent lowering of the GSL levels and, in turn, the GlcCer load into the lysosomes, may prevent such derangement. Studies on GlcCer synthase inhibitors able to pass the blood–brain barrier are in progress as novel therapeutic approaches [58]. In a cellular model of GBA deficiency, obtained by CRISP/Cas9 genome editing of HEK-293 cells, decreased ceramide levels were found to be associated with impaired secretory autophagy and intracellular α-synuclein accumulation [59], which is prevented by exogenous ceramide supplement or acid ceramidase inhibition. These results point out that the relative loss of GBA activity and consequent accumulation of GlcCer or its by-product GlcSph, or ceramide depletion, are pathogenic, impairing lysosomal function and autophagy [60] and, in turn, triggering the development of PD. Moreover, GBA deficiency may promote the spread of protein aggregates through extracellular vesicles [61]. On the other hand, other data suggests that GBA activity and GlcCer/GlcSph/ceramide levels are not necessary to determine the increased risk of developing PD. Carriers of the E326K GBA variant, which maintain substantial enzymatic activity, present increased risk of developing PD, although such mutation does not determine GD in the homozygotes [62]. Impaired autophagy due to GBA mutations was suggested to occur and to affect the clearance of damaged mitochondria as a consequence of altered proteostasis [50,63]. In mouse and cellular models carrying the L444P GBA variant, the loss of lysosomal enzyme activity blocks the degradation of autophagic cargos, whereas the variant GBA protein impairs autophagy induction and the priming of damaged mitochondria, indicating a gain of toxic function for the mutant protein. This effect may be attributed to the ER retention of the variant GBA protein, resulting in an unfolded protein response [64]. In a mouse model, overexpression in the striatum of the N370S GBA variant affects α-synuclein and, in particular, its secretion through exosomes, while the simple inhibition of enzyme activity by conditurol-β-epoxide does not [65].

Enzyme replacement and substrate reduction therapies are currently approved and used in clinical practice for the treatment of GD patients suffering visceral symptoms of the disease [66]. They are ineffective in neuronopathic GD because they are unable to cross the blood–brain barrier. In light of the emerging complexity of GD pathogenesis, the above-mentioned mechanisms of disease also provide new potential targets of therapeutic approaches [66,67]. In particular, heterogeneity in the clinical outcome between patients harboring the same genotype introduced the concept of genetic modifiers of the disease. They are defined as genes able to alter the clinical phenotype of a disease, modifying the penetrance, expressivity, dominance, or pleiotropy of the causative defect. Several have been identified for GD, some acting directly on the GBA pathway (GBA2, SCARB2, UGCG), and others regulating lysosomal function (TFEB, transcription factor EB) or downstream pathways [68]. Small molecules able to cross the blood–brain barrier are able to increase TFEB activity, inhibit UGCG [58], or restore autophagy [69], representing promising examples of such novel drugs.

## 3. Other Diseases of Sphingolipid Metabolism Determining or Predisposing Individuals to Neurodegenerative Disorders

The growing and converging evidence supporting lysosome involvement in PD and the relevance of GBA variants as the main genetic risk factors for developing the disease have prompted several researchers to study potential connections between other lysosomal diseases and neurodegenerative disorders (data are summarized in Table 1). The first association was proposed between sphingomyelinase SMPD1 variants, causing Niemann–Pick disease types A and B and PD [70], and received further confirmations suggesting that reduced sphingomyelinase activity led to α-synuclein accumulation [71]. Niemann–Pick disease type C is clinically similar to Niemann-Pick disease type A, but is determined by mutations of a different gene (*NPDC1*, neural proliferation differentiation and control protein 1), coding a protein regulating endocytic transport in late endosomes and lysosomes. Interestingly, inhibition of GBA2 improves lysosomal function in fibroblasts from Niemann-Pick disease type C patients [72]. Thus, the question rose as to whether mutations of other genes responsible for known lysosomal storage diseases predispose patients to PD. Analysis of a large whole exome sequencing dataset available for PD [73] suggested the association of the disease with three proteins responsible for lysosomal diseases in addition to GBA and SMPD1. They include ASAH1 (acid ceramidase, causing Farber disease), CLN10 (cathepsin-D, a lysosomal aspartyl proteinase causing neuronal ceroid lipofuscinosis), and SLC17A5 (sialin, causing Salla disease). The latter is involved in the transport of the sialic acid residues released by the action of sialidases on gangliosides and glycoproteins out of lysosomes [74]. Of note, Farber disease is a typical spectrum disease where patients carrying the same mutation present very different clinical features (reviewed in [29]) (Table 1). Neuronal ceroid lipofuscinosis is a neurodegenerative disorder determined by the mutations of at least 13 different genes, all somewhat involved in autophagy [75]. Recent studies on α-galactosidase A (GLA) deficiency, causing Fabry disease, an X-linked disorder causing systemic symptoms, have suggested a possible association between GLA mutations and PD or multiple sclerosis [38,76]. Moreover, GLA activity was reported to be lower in PD cases compared to controls [77]. Similarly, galactocerebrosidase (GALC) mutations, causing Krabbe disease, are studied as potentially being related to neurodegeneration/multiple sclerosis and synucleopathies [78,79,80]. In this regard, lysosphigolipids (Figure 3) formed in the disease, and even in GD, Fabry disease, Niemann-Pick disease, and GM1 gangliosidosis (Table 1), were reported to affect endolysosomal transport and pH, leading to the possible formation of α-synuclein aggregates [81,82]. Signs of Parkinsonism are now considered to be detectable in various inherited metabolic disorders in addition to GD and Niemann Pick disease [83]. Recent studies highlight the potential link between PD and sphingolipids, particularly gangliosides. They were found to promote α-synuclein aggregation in vitro [84]. In cellular models, accumulation of gangliosides due to reduced activity of GBA variants prevented the formation of stable α-synuclein tetramers, and pharmacologic (miglustat) inhibition of GSL biosynthesis restored α-synuclein stabilization [85]. Reduced expression of galactosyltransferase B3GALT4 and sialyltransferase ST3GAL2, as detected by in situ hybridization, was found to occur specifically in neuromelanin producing neurons of the brains of PD patients [86]. Plasma levels of GM3 were found to be increased in PD patients [87].

It is also interesting to consider the data now available from inherited diseases of sphingolipid metabolism very recently reported (Table 2 and Table 3). In the defects of serine palmitoyl transferases *SPTLC1-2* and dihydroceramide desaturase *DEGS1*, causing forms of hereditary sensory neuropathy type 1 and hypomyelinating leukodystrophy, respectively, the presence of potentially toxic compounds is suspected to be involved in the disease pathogenesis. In particular, deoxysphingolipids formed by SPTLC1-2 variants were found to exert mitochondrial toxicity [10], and increased amounts of reactive oxygen species (ROS) were detected in fibroblasts from patients carrying DEGS1 variants. Interestingly, pharmacological inhibition of CERS prevents ROS accumulation [21].

Heterozygous variants of sphingomyelin synthase SGMS2, an enzyme coding a plasma membrane form of sphingomyelin synthase, were found associated with the clinical picture of Osteoporosis with Skeletal Dysplasia. Interestingly, the main clinical signs are restricted to the bones, but a spectrum of mild or very mild neurologic symptoms were detected. Disease severity appeared dependent on mislocalization of enzyme variants more than on levels of residual activity [23].

Together, these data suggest that sphingolipid metabolism interacts with cellular homeostasis through several mechanisms that can be affected by enzyme mutations, as recapitulated by the ability of GBA variants to determine a spectrum disease, including neurodegenerative disorders such as PD.

## 4. Specific Diseases of Ganglioside Biosynthesis Determining Neurodegenerative Disorders

### 4.1. GM2 Synthase Deficiency (B4GALNT1-CDG), HSP26, and Other HSPs

B4GALNT1 is the enzyme necessary to extend all ganglio-series gangliosides [88,89] (Figure 2). Consequently, its impairment leads to a lack of complex gangliosides and accumulation of GM3 and GD3. Twelve pathogenic variants of the gene have been reported so far in 12 families, involving 38 individuals, all showing a form of complex HSP previously referred to as HSP26 [90,91,92]. The syndrome is characterized by late onset of motor impairment of the legs accompanied by mild to moderate cognitive impairment, sometimes associated with psychiatric illness and/or non-neurological symptoms such as pes cavus or hypogonadism in males (Table 3). Very recently [93], it has been reported that nine out of 11 tested mutations lead to a complete loss of enzyme activity, while two variants (R300C and R228*) maintain a small but detectable residual activity in vitro. Three variants appeared not detectable by western blot, while the other eight were found to be correctly localized to the Golgi apparatus by confocal microscopy, suggesting that the loss of function is the main pathogenic event. This hypothesis is also supported by the evidence that b4galnt1 KO mice present a similar phenotype [94], although in some but not all cases [95]. However, HSP is due to the defect of a plethora of different genes [34]. Two of them directly involve sphingolipid metabolism in addition to *B4GALNT1*: *GBA2*, responsible for HSP46 [28], and *FA2H*, involved in HSP35 [96] and coding for fatty acid 2-hydroxylase [97]. Interestingly, HSP11 is due to the loss of function of spatacsin, a product of the *SPG11* gene, which is involved in lysosome membrane recycling. In the spg11 KO mouse model [98], loss of spatacsin impairs ganglioside clearance from the lysosome and determines the accumulation of autophagy markers. In addition, neurodegeneration was stimulated by the accumulation of gangliosides in cultured neurons and was prevented by miglustat. The same authors were also able to reduce motor impairment by inhibiting ganglioside biosynthesis in the HSP11 zebrafish model.

### 4.2. GM3 Synthase Deficiency (ST3GAL5-CDG)

ST3GAL5 displays unique GM3 synthase activity, being responsible for the biosynthesis of ganglio-series gangliosides of the a-, b-, and c-series, with the exception of 0-series gangliosides, such as GM1b and GD1α [99] (Figure 2). Five different pathogenic mutations have been reported so far, all causing a very severe syndrome characterized by normal pregnancy and delivery but early onset of neurological symptoms including drug-resistant epilepsy, failure to thrive, and impaired hearing and vision. Growth delay and progressive regression occur, giving rise to deafness, blindness, and general motor and cognitive impairment [25,100]. While the original description of a few families suggested a potential different degree of pathogenicity between mutations [101,102,103], a recent survey of several cases affected by the same R288* non-sense mutation indicated the opposite [104]. Some of these patients lack clinical seizures or were initially able to crawl, as originally reported only for two siblings carrying the compound C195S/G201A mutations [103]. Accordingly, we have recently shown that all reported mutations abolish any detectable enzyme activity, without differences between the single mutations [100]. Moreover, we also found a normal localization of the variant ST3GAL5s. Altogether, this data is in agreement with the current hypothesis that the total absence of gangliosides is responsible for the general lack of neuron homeostasis, leading to inflammation and, consequently, loss of several functions [105]. However, many patients present mildly elevated blood lactate and their cells have an impaired respiratory chain function [25,100]. In this context, the hypothesis that impaired autophagy and, in particular, mitophagy may be involved in the disease pathogenesis should be kept in mind. The enormous distance between the devastating human syndrome and the very mild KO mouse model [106,107] corroborates such a suggestion.

### 4.3. ST3GAL3-CDG, Non-Syndromic Intellectual Disability and West Syndrome

ST3GAL3 is considered the enzyme responsible for the α2,3 sialylation of galactose preferentially β1,3 linked to GlcNAc in several glycoconjugates, including glycoproteins carrying both N- and O- glycans and GSLs of the lacto-series [108,109]. Data obtained in vitro with the mouse enzyme suggested a strong preference for the Galβ1,3GlcNAc acceptor sequence [110], ruling out the ability to use the Galβ1,3GalNAc sequence, including ganglio-series gangliosides. Conversely, double st3gal2/st3gal3 KO mice exhibited substantial loss of GD1a and GT1b gangliosides in their brain, not found in single st3gal2 KO animals, suggesting the ability of the enzyme to use the Galβ1,3GalNAc sequence of GSLs in vivo [111]. Detailed data are not available for the human enzyme, which is considered involved in the α2,3 sialylation of the Galβ1,3GlcNAc sequence necessary for constructing the tetrasaccharide epitope of the CA19.9 antigen [112], an adhesion molecule potentially involved in cancer [113,114].

Two pathogenic variants of ST3GAL3 were found in adult members of two families reported to suffer from non-syndromic autosomal recessive intellectual disability [115]. The A13N variant appeared to be associated with preserved enzymatic activity in vitro and even only partial retention of the protein in the ER.

Conversely, the other mutation, N370T, was found to determine total loss of enzymatic activity together with substantial retention in the ER. Further, a third mutation (A320P) was reported in four infants from a consanguineous family that were diagnosed with West syndrome, evolved to Lennox–Gestaut syndrome [35], which are age-dependent epileptic encephalopathic syndromes defined by a specific electroencephalography pattern and associated with developmental arrest or regression. Such a mutation provided a complete loss of enzymatic activity and impaired protein localization [116].

Recently, neurons differentiated from iPSc cells obtained from fibroblasts carrying the A320P mutation [116] were studied in order to find the glycoproteins lacking sialyl-α2,3 residues, as assessed by *Maackia amurensis* labeling. A defect in a single 80-kDa glycoprotein was detected in differentiated cortical neurons, together with altered adhesion properties. In the st3gal3 KO mouse model of the disease, the animals lack neurologic symptoms, presenting only mild immunologic anomalies [117,118]. In this case, retention in the ER of the variants suggests that they could be misfolded, irrespective of the catalytic activity maintained, and this would be compatible with a pathogenic role of altered proteostasis, irrespective of the lack of sialylation, on either glycoproteins or glycosphingolipids.

## 5. Conclusions

Recent studies on the GD/PD relationship demonstrate that GBA variants predispose individuals to PD through molecular mechanisms that have the potential to operate in other disorders affecting sphingolipid metabolism, leading in turn to neurodegeneration.

One such mechanism depends on the amounts of individual molecules accumulated. It has been well proven in the case of the inflammatory role played by GlcCer through the activation of complement in the tissues affected by type 1 GD [45]. This is also potentially relevant in conditions where gene mutations predispose individuals to the accumulation of non-physiologic by-products, such as lysosphingolipids in several diseases. (Table 1), deoxysphingolipids in hereditary sensory neuropathy type 1, and Δ14-cis sphingolipids in defective desaturase activity (Table 2). Similarly, a direct pathogenic effect is possibly due to the lack of specific products in the diseases affecting biosynthetic enzymes, as typically seen in ST3GAL5 deficiency.

Another relevant mechanism suggested by the GBA/PD relationship involves the traffic of vesicles towards lysosomes, affecting autophagy. The peculiar role of autophagy in the survival of neurons is well known, as well as the pathogenic role of its impairment in neurodegeneration [119]. In PD, α-synuclein accumulation appears strongly dependent on autophagy, as detailed in the section above. Sphingolipid metabolism strongly interplays with neuronal autophagy, with particular relevance of gangliosides, ceramide, SM, S1P, and GlcCer. Autophagy is impaired by increased GlcCer and SM levels, as determined by GBA and sphingomyelinase SMPD1 deficits, but stimulated by increased ceramide levels (reviewed in [120]) or impaired by reduced levels [59]. Some HSPs are due to impaired sphingolipid metabolism, others to defective mitochondrial proteins, and others to gene mutations that code proteins involved in organelle trafficking and shape [34]. One of the latter, spastin, is considered particularly relevant in the disease pathogenesis suggesting that toxic gain-of-function mechanisms operate in a context of a nervous system made vulnerable by haploinsufficiency [121]. A reasonable hypothesis is that any imbalance between the amounts of individual sphingolipids may impair endocytic trafficking, affecting cargo load or autophagy directly, as proven for GBA variants. In this regard, it is important to note that some models revealed specific impairment of mitophagy in PD [64]. In ST3GAL5-CDG, the lack of ganglio-series gangliosides is probably accompanied by the accumulation of globosides, and mitochondrial defects are present, sometimes reported to misdirect the diagnosis [122], although brain mitochondria lack gangliosides [123]; we speculate that impaired mitophagy may occur in the disease as suggested for lysosomal storage diseases [124].

Finally, GBA variants affect cell homeostasis through altered proteostasis due to ER stress or other consequences of protein misfolding [39,48,54]. In ST3GAL3-CDG, protein variants were reported to be improperly localized and partially retained in the ER.

A growing number of pharmacologic inhibitors of sphingolipid metabolism are available and studied as drugs potentially suitable in human diseases [125]. Once those able to cross the blood–brain barrier are selected, it will be possible in the future to use them to target the pathogenic mechanisms of some neurodegenerative disorders, possibly in association with the substances potentially able to affect lysosomal function, autophagy, and proteostasis [68,69,126].

## Figures and Tables

**Table 1:** Enzymes involved in sphingolipid degradation and recycling.

Enzyme	Hugo Symbol	Subcellular Site	Disease	Main Clinical Features	Biochemical Features	Ref
Acid ceramidase	ASAH1	Lysosome	Farber disease and spinal muscular atrophy with progressive myoclonic epilepsy	Typical spectrum disease varying from the classic triad of subcutaneous nodules, joint contractures, and hoarse voice to moderate or severe forms involving hematopoietic, gastrointestinal, respiratory, and neurologic symptoms, including seizures; developmental delay and death in the early childhood.	The same Y137C mutation provided very mild phenotype in a patient and severe neurologic phenotype in another. Two SNPs are associated with schizophrenia. Residual activity >5% is associated with survival. Candidate risk factor for Parkinson´s disease (PD).	[29,73,127]
Neutral ceramidase	ASAH2	Plasma membrane	None reported	Main expression in the small intestine and colon, probable role in digestion.		[128]
Alkaline ceramidases	ACER1	Endoplasmic reticulum (ER)		Main expression in the skin.		[129]
	ACER2	Golgi apparatus				
	ACER3	ER and Golgi apparatus	Progressive leukodystrophy	Developmental regression at 6–13 months, starting with peripheral neuropathy and leading to severe dysmorphic facial feature and psychomotor impairment, requiring mechanical ventilation.	Plasma accumulation of ceramides, dihydroceramides, glucosylceramide (GlcCer), and lactosylceramide (LacCer). Increased blood lactate levels.	[30]
Sphingosine kinases	SPHK1	Plasma membrane (main)	None reported			[130]
	SPHK2	ER (main)				
Sphingosine lyase	SGPL1	ER	Syndromic steroid-resistant nephrotic syndrome	Steroid-resistant nephrotic syndrome with facultative ichthyosis, adrenal insufficiency, immunodeficiency, and neurological defects.	Reduced activity and protein mislocalization are frequent between mutations. Ceramides are elevated in the conditioned culture medium of patient fibroblasts.	[131]
Galacto-cerebrosidase	GALC	Lysosome	Krabbe disease	Infantile onset (within 6 months). Increased irritability, spasticity, developmental delay along with unexplained fever, blindness, and deafness. Severe motor and mental deterioration.	Poor genotype–phenotype relationship. Galactosyl-sphingosine (psychosine) accumulates, affecting endolysosomal transport and pH. Aggregated forms of α-synuclein reported.	[37,49,79,91]
Gluco-cerebrosidases	GBA	Lysosome	Gaucher disease	Type 1 disease classically includes inflammatory signs in visceral organs that appear in adulthood; types 2 and 3 are instead neuronopathic, with early onset and progression at different rates. Recently proposed to be a spectrum disease. Main genetic risk factor for PD even in heterozygous carriers (see details in the text).	Poor genotype–phenotype relationship. GlcCer and glucosylsphingosine accumulate, affecting vesicle traffic and autophagy including mitophagy, which are also impaired by altered proteostasis. Strong evidence that glucocerebrosidase (GBA) variants affect α-synuclein accumulation (see details in the text).	[27,33,50,54,57]
	GBA2	Microsomes	HSP46/Cerebellar ataxia with late-onset spasticity	Early onset of motor impairment with mental retardation, cataract, and hypogonadism in males. MRI: cerebellar and corpus callosum atrophy.	Loss of enzymatic activity in almost all known mutations. Inhibition of activity in fibroblasts from Niemann–Pick patients restores endolysosomal pH.	[28,44,72]
β-galactosidase	GLB1	Lysosome	GM1 gangliosidosis	Infantile form: early onset and rapid progressive psychomotor deterioration, skeletal abnormalities, visceromegaly, and death. Juvenile and adult phenotypes characterized by slowly progressive neurological degeneration and mild skeletal changes.	Poor genotype–phenotype correlation. GM1, LacCer, and lactosylsphingosine accumulate causing impairment of endolysosomal transport and pH, autophagy, and mitochondrial function). ER stress detected.	[81,99]
Hexosaminidase A	HEXA	Lysosome	Tay–Sachs disease	Infantile form: early onset of neurodevelopmental dysfunctions, hypotonia and eye movement abnormalities. Progression includes dysphagia, seizures, macrocephaly, and death until age 3.5 years. Juvenile onset includes ataxia, dysarthria, dysphagia, progressive hypotonia, seizures, and death until 15 years of age.		[99]
Hexosaminidase B	HEXB	Lysosome	Sandhoff disease	Juvenile onset form: reduced attention, weakness, hypotonia, and progressive psychomotor impairment. Adult-onset form: milder phenotype due to residual enzymatic activity. Muscle weakness and motor symptoms.	GM2, asialo-GM2, and globoside accumulate. Deposits of α-synuclein reported.	[49,99]
GM2 activator	GM2A	Lysosome	GM2 gangliosidosis	Similar to Tay–Sachs disease.		[99]
α-galactosidase	GLA	Lysosome	Fabry disease	X-linked recessive, phenotypes from healthy to severe in women, severe to fatal in men. Various organs potentially involved, including peripheral and central nervous system. Cardiovascular involvement is frequent and at high risk for stroke and arrhythmias.	Globotriaosylceramide and globotriaosylsphingosine accumulate. Activity of respiratory chain enzymes reduced, protein trafficking and sorting altered, autophagy-lysosome pathway dysregulated. Impaired α-synuclein degradation.	[38,76,132]
Acid sphingomyelinase	SMPD1	Lysosome and secretory	Niemann–Pick disease types A and B	The gene is paternally imprinted. Type A: acute, early onset with failure to thrive and hepatosplenomegaly. Rapid and progressive neurodegenerative course, hypothonia and death until age of 3 years. Cherry-red spot in the macula. Type B: chronic, no neurologic signs. Hepato-splenomegaly and signs of liver failure. Impaired pulmonary function. High levels of serum triglycerides and LDL-cholesterol, low levels of HDL-cholesterol. Reddish brown or cherry red spot in the macula. SMPD1 variants are confirmed risk factor for PD.	Good genotype-phenotype correlation. Sphingomyelin and lysosphingomyelin (sphingosine-phosphocoline) accumulate. Increased levels of cholesterol, GlcCer, LacCer, and gangliosides. Decreased activity levels led to α-synuclein accumulation.	[22,36]
Neutral sphingomyelinases	SMPD2	Plasma membrane	None reported			[22]
	SMPD3	ER, Golgi apparatus, and nucleus				
	SMPD4	ER and Golgi apparatus				
	SMPD5	Mitochondria and ER				

**Table 2:** Endoplasmic reticulum resident enzymes involved in the initial biosynthesis of sphingolipids.

Enzyme	Hugo Symbol	Disease	Inheritance	Main Clinical Features	Biochemical Features	Ref.
Serine palmitoyl transferases	SPTLC1	Hereditary sensory neuropathy (Type 1)	Autosomal dominant	Onset of sensory impairment spanning the second to fifth decades, frequent motor impairment and burning pain episodes; distal to proximal progression. Mutations of either one or two subunits determine identical clinical phenotypes.	Alanine and glycine used instead of serine producing deoxysphinganine and deoxyceramide, which have mitochondrial toxicity in vitro.	[9,10]
	SPTLC2					
	SPTLC3	None reported				
3-keto-dihydro-sphingosine reductase	KDSR	Erythrokeratoderma or ichtyosis with anemia and thrombocytopenia	Autosomal recessive	No neurologic signs. Heterogeneous skin and hematologic symptoms; spontaneous remission with age in some cases.	Retinoic acid therapy effective, probably stimulating salvage pathway from sphingosine.	[11,12,13]
Dihydro-ceramide synthases	CERS1	Myoclonus epilepsy	Autosomal recessive	Ataxia at the age of one year, delay in development, generalized tonic–clonic seizures, action myoclonus with onset between 6 and 16 years of age. Cognitive deterioration up to dementia.Magnetic resonance imaging: brainstem atrophy.		[14,15]
	CERS2	Progressive myoclonus epilepsy	27 kb heterozygous deletion	Tonic–clonic seizures prevented by valproic acid, learning disability, progressive myoclonic epilepsy, moderate intellectually disability with dysarthria and ataxia.		[17]
	CERS3	Congenital ichthyosis	Autosomal recessive	No neurologic signs. Congenital ichthyosis characterized by collodion membranes at birth, generalized scaling of the skin, and mild erythroderma.	Specific loss of ceramides with acyl chains from C26 up to C34 in keratinocytes.	[16]
	CERS4	None reported				[18]
	CERS5					
	CERS6					
Dihydro-ceramide desaturases	DEGS1	Hypomyelinating Leukodystrophy	Autosomal recessive	Onset at 0.5–24 months. Failure to thrive, developmental delay, epilepsy, neurogenic muscular atrophy, severe motor arrest, microcephaly, dystonia and severe spasticity. MRI: hypomyelination, thin white matter, progressive thalamic and cerebellar atrophy.	Presence of Δ14-cis sphingolipids; inhibition of CERS ameliorates phenotype in zebrafish model, and reactive oxygen species (ROS) levels in patient fibroblasts.	[20,21]
	DEGS2	None reported		Relevant in stratum corneum.	Involved in the metabolism of sphingolipid containing 4-hydroxysphingosine (phytosphingosine).	[19]
SM synthase related protein	SAMD8	None reported			Involved in the synthesis of ceramide phosphoethanolamine.	[22]
GalCer synthase	UGT8	None reported				

**Table 3:** Golgi apparatus resident enzyme involved in the biosynthesis of complex sphingolipids. Note that sphingomyelin synthase SGMS2 resides in the plasma membrane.

Enzyme	Hugo Symbol	Disease	Inheritance	Main Clinical Features	Biochemical Features	Ref.
Sphingomyelin synthases	SGMS1	None reported				[22]
	SGMS2 (plasma membrane resident)	Osteoporosis with skeletal dysplasia	Autosomal dominant	Minor neurologic signs detectable in some cases. Childhood onset osteoporosis with or without cranial sclerosis, neonatal fractures, short stature, and spondylometaphyseal dysplasia.	Variants are frequently mislocalized or retained in the ER; catalytic activity maintained by some variants.	[23]
Glucosylceramide synthase	UGCG	Congenital ichthyosis	Autosomal recessive	Normal growth parameters at birth, but covered with a collodion membrane; death at age 2 weeks because of severe hypernatremic anuric renal failure.	Phenotype similar to that of the keratinocyte-conditional KO mouse.	[24]
UDP-Gal: GlcCer β1,4-galactosyltransferase	B4GALT6	None reported			Synthesizes lactosylceramide.	
UDP-Gal: lactosylceramideα-1,4-galactosyl-transferase	A4GALT				Synthesizes globotriosyl ceramide.	
UDP-GlcNAc: lactosylceramide β-1,4-GlcNAc transferase	B3GNT5				Synthesizes lacto-N-triosyl ceramide.	
GM3 synthase	ST3GAL5	ST3GAL5-CDG	Autosomal recessive	Normal at birth, early onset severe neurological signs. Failure to thrive, regression, severe hearing, visual, motor, and cognitive impairment (see details in the text).	Mitochondrial defects in patients. Globosides accumulate in human fibroblasts.	[25,100,101,102,103,104]
GM2/GD2/GA2 synthase	B4GALNT1	Hereditary spastic paraplegia 26 (B4GALNT1-CDG)	Autosomal recessive	Late onset motor impairment of the legs accompanied by mild to moderate cognitive impairment, sometimes associated with psychiatric illness and/or non-neurological symptoms (see details in the text).	GM3 and GD3 accumulate in vivo and in vitro models.	[90,91,92]
UDP-Gal: GM2/GD2/GA2 β1,3-galactosyltransferase	B3GALT4	None reported			Synthesizes gangliosides GM1, GD1a, and GD1b.	
CMP-Sial: GlcNAcβ1,3(4) sialyltransferase	ST3GAL3	Non syndromic autosomal recessive intellectual disability/West syndrome	Autosomal recessive	Only intellectual disability reported when diagnosed in adults, West syndrome when diagnosed in early childhood (see details in the text).	ER retention frequent in variants, enzyme activity maintained in one variant.	[35,115,116]

## Abbreviations

CDG	Congenital disorders of glycosylation
ER	Endoplasmic reticulum
GalCer	Galactosylceramide
GD	Gaucher disease
GlcCer	Glucosylceramide
GlcSph	Glucosylsphingosine
GlcNAc	N-acetylglucosamine
GSL	Glycosphingolipid
HSP	Hereditary spastic paraplegia
LacCer	Lactosylceramide
PD	Parkinson disease
ROS	Reactive oxygen species
SCARB2	Scavenger receptor class B member 2/lysosomal integral protein 2 (LIMP2)
SM	Sphingomyelin
S1P	Sphingosine-1-phosphate
